# Longitudinal study of diabetes prevalence and hospitalisations among care experienced and general population children in Scotland: evidence of an end of care “cliff edge”?

**DOI:** 10.23889/ijpds.v7i3.2000

**Published:** 2022-08-25

**Authors:** Robin Flaig, Jacqui Oakley, Kirsteen Campbell, Katharine Evans, Stela McLachlan, Richard Thomas, Emma Turner, Andy Boyd

**Affiliations:** 1 University of Edinburgh; 2 University of Bristol

## Objectives

The UK Longitudinal Linkage Collaboration (UK LLC) is a new, unprecedented infrastructure enabling research into the COVID-19 pandemic. The UK LLC integrates data from >20 UK longitudinal studies with systematically linked health, administrative and environmental records to facilitate cross-disciplinary COVID-19 research for accredited UK based researchers.

## Approach

Bringing together all of the key components that form the UK LLC was a huge challenge that may have only been possible in the midst of the pandemic. First, we collaborated with the Longitudinal Population Studies (LPS) to create and agree how data linkage, data provision and applications to access the UK LLC would work. In parallel, public contributors helped to create fair processing materials. Finally, we worked closely with NHS Digital and other key national data providers to organise approvals for all studies to be linked, and for the UK LLC to have delegated decision-making for research applications.

## Results

We faced a myriad of challenges creating the UK LLC including:

- Short timeframe and short-term funding structure – initial funding for six months with an 18-month extension.
- Working across >20 different LPS and four nations with different structures for access, consent and data provision.
- Lack of capacity at various points in the data pipeline due to the volume of COVID-19 research required and underway across the involved organisations.
- Data processing complexities – split data method means no one can see the entire process therefore catching linkage errors requires working across four different organisations.
- With such complex data flows it is challenging to find the balance with communications about data to the public – being accurate about what we are doing, but expressing the complexity in lay terms.

## Conclusion

Creating the UK LLC required collaboration with LPS, data providers and researchers. An iterative approach to creating the data application and data provision pipelines was crucial in developing these processes. The UK LLC was built quickly, from initial funding in October 2020 to provisioning data to researchers in December 2021.

